# Predicting the right! Validation of right heart failure predictive risk models after primary durable ventricular assist device implantation

**DOI:** 10.1371/journal.pone.0357025

**Published:** 2026-09-09

**Authors:** Marjolijn Carolien Sales, Rachad Zayat, Nima Hatam, Thomas Berg, Lachmandath Tewarie, Ajay Moza, Natasja Wanita Monisha Ramnath

**Affiliations:** Department of Cardiac Surgery, University Hospital RWTH Aachen, Aachen, Germany; University of Naples Federico II, ITALY

## Abstract

**Objectives:**

Perioperative right heart failure remains a severe complication after durable left ventricular assist device implantation. Accurate preoperative risk assessment is crucial to identify patients requiring advanced medical or mechanical biventricular support. Existing predictive risk models show limited comparability and lack consistent external validation. We aimed to validate commonly used right heart failure risk models using a standardized definition and, as a secondary exploratory analysis, to evaluate a parsimonious Firth-penalized model in our cohort.

**Methods:**

We performed a single-center, retrospective observational study of 105 patients undergoing durable left ventricular assist device implantation from April 2015 to February 2023. Right heart failure was defined according to the Interagency Registry for Mechanically Assisted Circulatory Support definition. Preoperative EUROMACS, Michigan, CRITT, BiVAD, Utah, and a modified Utah score were calculated. The postoperative EUROMACS score, which includes cardiopulmonary bypass time, was analyzed separately as an early perioperative risk marker. Discrimination was assessed using receiver-operating characteristic analysis. The exploratory five-variable Firth model was internally validated by bootstrap optimism correction and calibration analysis.

**Results:**

Moderate-severe right heart failure occurred in 21 patients (20.4%). Among established scores, AUC values ranged from 0.394 (Utah) to 0.729 (postoperative EUROMACS). The exploratory five-variable Firth model, including INTERMACS profile, total bilirubin, aspartate aminotransferase, diastolic pulmonary artery pressure, and pulmonary vascular resistance, showed an apparent AUC of 0.854. After 2,000 bootstrap resamples, the optimism-corrected AUC was 0.773, with an optimism-corrected Brier score of 0.149 and calibration slope of 0.586, indicating relevant optimism and potential overfitting.

**Conclusions:**

Established right heart failure risk scores showed limited-to-moderate discrimination in this cohort. Postoperative EUROMACS achieved the highest AUC but should not be interpreted as a purely preoperative prediction tool. The exploratory Firth model identified a potentially informative combination of clinical status, hepatic laboratory markers, and pulmonary hemodynamics, but remains hypothesis-generating and requires external validation before clinical implementation.

## Introduction

Heart failure is a major global health concern, and its burden is expected to increase even further in the future [1]. In end-stage heart failure, the implantation of a durable left ventricular assist device (LVAD) has emerged as a standard of care [2], especially as device technologies have advanced considerably over the past years [3,4]. Despite favorable outcomes in the current era of fully magnetically levitated devices with improved survival rates and lower incidences of adverse events such as bleeding and thromboembolism [5], other issues have not yet been overcome. Perioperative right heart failure (RHF) remains a challenging and severe complication following LVAD implantation. Its incidence has been reported to range between 4% and 50% [6] based on varying definitions and diagnostic approaches for RHF. Patients with perioperative RHF have been shown to exhibit higher morbidity and mortality [7,8]. The rate of adverse events as well as mortality increases further, when right-sided mechanical circulatory support (MCS) is required [9,10]. Current reviews suggest that extent and timing of right ventricular support may play an important role in patient outcomes [11,12], while other studies report no differences between concomitant and secondary right-sided MCS [9]. As these outcomes may be preventable through appropriate patient selection and tailored strategies, accurate preoperative diagnosis and risk assessment for RHF are crucial for identifying patients suitable for isolated LVAD implantation or patients at risk and in need for advanced medical and/or mechanical biventricular support. Over the past years, numerous scoring systems to predict RHF in LVAD patients have been published [6,8,13–19]. Most of these scores rely on variably weighted multimodal preoperative diagnostics including clinical data, laboratory findings, echocardiography and invasive hemodynamic measurements, while others attempt to simplify prediction based solely on laboratory values [19] or echocardiography [16]. All currently available RHF predictive risk models show limited comparability due to derivation from small cohorts, heterogeneity in LVAD patients and treatment protocols as well as inconsistent definitions for RHF, and a lack of external validation. Therefore, we aimed to validate commonly used RHF predictive risk models using a standardized INTERMACS-based definition for RHF and to explore whether a parsimonious multimodal model combining clinical status, laboratory markers, and hemodynamic parameters could identify candidate predictors for future validation.

## Patients and methods

### Study design

We performed a single-center, retrospective observational study of 105 consecutive adult patients scheduled for isolated durable LVAD implantation at our clinic from April 2015 to February 2023. Perioperative clinical, laboratory, echocardiographic, and hemodynamic data were obtained. RHF was defined according to INTERMACS (Interagency Registry for Mechanically Assisted Circulatory Support) Appendix A – Adverse Event Definitions, Version 10/11/2021. Two patients were subsequently excluded, as the definition of RHF could not be determined due to intraoperative death from causes other than RHF. The resulting groups of none-mild RHF (n = 82) and moderate-severe RHF (n = 21) were compared.

Our study was conducted in accordance with the ethical standards of the Declaration of Helsinki and was approved by the independent medical ethics committee of RWTH Aachen University Hospital, Aachen, Germany (EK 23–247). Due to the retrospective nature of the study, the requirement for informed consent was waived by the ethics committee. Data were accessed for research purposes after ethical approval was granted on 1 August 2023. The authors had access to identifiable clinical data during data collection and analysis; data were handled according to institutional privacy requirements.

### Data acquisition

All data were obtained from both electronic and/or paper-based patient records and were collected in an electronic case report file using REDCap (Research Electronic Data Capture, hosted at Vanderbilt University, Nashville, TN, USA). Clinical data included basic demographics, comorbidities, etiology, history and current stage of cardiac disease, medications, preoperative treatments, need for organ support (e.g., ventilation, dialysis, mechanical circulatory support), the requirement for continuous intravenous inotrope (epinephrine, milrinone, dobutamine, and/or levosimendan) or vasopressor (norepinephrine, vasopressin) therapies, and surgical parameters. Body mass index (calculated as weight/height^2^) and body surface area (calculated using the Dubois formula) were determined. INTERMACS class was recorded as reported or was retrospectively classified based on clinical data if not included in the written surgical report.

Laboratory data were obtained preoperatively as close as possible to the time of surgery and included a full blood count, coagulation parameters, cardiac enzymes, and renal and liver function tests.

Echocardiographic evaluations closest to the time of surgery were analyzed for heart chamber diameters, left ventricular ejection fraction, tricuspid annular plane systolic excursion (TAPSE), tricuspid annular systolic velocity, visually graded right ventricular function (none, mild, moderate, severe), and inferior vena cava enlargement > 20 mm.

Hemodynamic data were obtained either during right heart catheterization or in the intensive care unit using a Swan-Ganz catheter. Monitoring included heart rate, systemic blood pressures, central venous pressure, right atrial (RA) pressure (if available, otherwise equated with central venous pressure), pulmonary artery (PA) blood pressures, and pulmonary capillary wedge pressure (PCWP). Cardiac output was measured either by Fick principle (in the catheterization lab) or by thermodilution (using a Swan-Ganz catheter). Pulmonary artery pulse pressure (systolic PA pressure – diastolic PA pressure), transpulmonary gradient (mean PA pressure – PCWP), diastolic pulmonary gradient (diastolic PA pressure – PCWP), cardiac index (cardiac output/body surface area), stroke volume (cardiac output/heart rate), pulmonary vascular resistance (transpulmonary gradient/cardiac output), systemic vascular resistance ((mean arterial pressure – central venous pressure)/cardiac output), right atrial to pulmonary capillary wedge pressure ratio (RA pressure/PCWP), pulmonary artery pulsatility index ((systolic PA pressure − diastolic PA pressure)/ RA pressure), right ventricular stroke work index (stroke volume × (mean PA pressure – central venous pressure)/body surface area), pulmonary artery compliance (stroke volume/PA pulse pressure), pulmonary artery elastance (systolic PA pressure/stroke volume), and the TAPSE to systolic PA pressure ratio (TAPSE/systolic PA pressure) were calculated. Mixed and/or central venous oxygen saturation were also recorded.

Surgical information included the intention of treatment, application and model of LVAD, surgical access route, cardiopulmonary bypass time, cross-clamp and reperfusion times (if applicable), and any concomitant procedures.

Postoperative outcomes and mortality were recorded. We analyzed major adverse events, organ replacement therapies, need for inotropic support, the need for inhaled nitric oxide, duration of intensive care unit and hospital stays, in-hospital mortality due to right heart failure or other causes, 30-day survival, and long-term survival.

### Right heart failure predictive risk models

We selected commonly used predictive risk scores for RHF that are well described in the literature and applied them to our study population: the preoperative EUROMACS (European Registry for Patients with Mechanical Circulatory Support) RHF risk score [6], Michigan Right Ventricular Failure (RVF) risk score [16], CRITT (central venous pressure, right-ventricular dysfunction, intubation, tricuspid valve regurgitation, tachycardia) score [13], BiVAD (biventricular assist device) score [15], and Utah RVF risk score [14]. We also calculated the postoperative EUROMACS score [6]. Because this score includes cardiopulmonary bypass time, which is only available after LVAD implantation, it was evaluated separately as an early perioperative/dynamic risk marker and was not considered directly comparable to purely preoperative scores. All scores were calculated according to their respective original publications (S1 Table). In case of missing data for individual score variables, we still evaluated the predictive risk for RHF wherever an unambiguous classification was possible.

The Utah RVF risk score includes preoperative dependency on an intra-aortic balloon pump as a predicting parameter. Because extracorporeal membrane oxygenation and/or Impella® (Abiomed, Inc., Danvers, MA, USA) are now frequently used as temporary MCS instead of intra-aortic balloon pump support, we created a modified Utah score grouping these temporary MCS devices together. This modification has not been externally validated and was therefore analyzed only as an exploratory sensitivity analysis.

### Statistical analysis

Continuous variables were inspected for distributional characteristics and summarized as mean ± standard deviation or median with interquartile range as appropriate. Categorical variables were reported as counts and percentages. Missing values were neither replaced nor imputed; complete-case datasets were used for multivariable and bootstrap analyses. Group comparisons between patients with and without moderate–severe postoperative right heart failure were performed using the Mann–Whitney U test for continuous variables and χ² or Fisher’s exact test for categorical variables.

Variables associated with moderate–severe RHF at a significance level of p < 0.05 in univariate analysis or judged to be clinically relevant based on prior literature (right-sided or pulmonary hemodynamics, markers of end-organ congestion, and preoperative clinical status) were considered candidate predictors and entered into an exploratory full multivariable model. The full list of univariate associations and the exploratory full model are provided in the Supporting information. The purpose of this modelling step was hypothesis generation rather than derivation of a clinically ready prediction score.

Given the limited number of RHF events (n = 21) and the risk of model overfitting, multicollinearity, and small-sample bias, we adopted a structured multivariable modelling strategy. First, all candidate variables with p < 0.05 in univariate testing were simultaneously entered into an exploratory full multivariable logistic regression to assess parameter stability, collinearity, and model convergence. To avoid redundancy among strongly correlated hemodynamic indices (e.g., cardiac output/index, stroke volume/index, systolic/mean pulmonary pressures), only one representative variable per physiological domain was selected. INTERMACS profile was included as a key indicator of preoperative acuity, while total bilirubin and aspartate aminotransferase represented hepatic congestion, diastolic pulmonary artery pressure represented pulmonary vascular loading, and pulmonary vascular resistance reflected afterload to the right ventricle.

Because standard logistic regression showed separation tendencies and produced convergence warnings — reflecting the small event-to-variable ratio — final modelling was performed using Firth bias-reduced penalized logistic regression, which is recommended for rare events and small samples to reduce small-sample bias and improve estimate stability. The final parsimonious exploratory model included the five most clinically informative and statistically stable predictors: INTERMACS profile, total bilirubin, diastolic pulmonary artery pressure, aspartate aminotransferase, and pulmonary vascular resistance. Adjusted odds ratios (ORs) with 95% confidence intervals (CIs) were derived. This model was not intended to represent a validated clinical score.

To evaluate discriminative performance, receiver-operating characteristic (ROC) curves were generated for each established RHF prediction score (preoperative EUROMACS, postoperative EUROMACS, CRITT, Michigan, BiVAD, Utah, and modified Utah), for INTERMACS profile alone, and for the exploratory multivariable Firth model. The area under the ROC curve (AUC) with 95% CIs was used to quantify discrimination. Comparative ROC curves are presented in a two-panel figure.

To assess internal validity of the exploratory five-variable Firth model, bootstrap optimism correction was performed using 2,000 bootstrap resamples. In each bootstrap sample, the final fixed Firth-penalized logistic regression model was refitted using the same five predictors: INTERMACS profile, total bilirubin, aspartate aminotransferase, diastolic pulmonary artery pressure, and pulmonary vascular resistance. Model performance was evaluated both in the bootstrap sample and in the original complete-case dataset. Mean optimism was calculated as the difference between bootstrap-sample and original-sample performance and was subtracted from the apparent performance estimate. Discrimination was assessed using AUC. Calibration was assessed by calibration intercept, calibration slope, Brier score, and graphical calibration. Because of the limited event number, calibration intercept and slope were estimated using Firth logistic recalibration.

Overall survival was analyzed using Kaplan–Meier methods, with time defined as days from LVAD implantation to death or censoring at last known follow-up. Patients were stratified by postoperative RHF severity (none–mild versus moderate–severe). Differences between groups were evaluated using the log-rank test. Unadjusted associations between RHF severity and mortality were quantified using Cox proportional hazards regression, reporting hazard ratios (HRs) with 95% CIs. Model assumptions were assessed using Schoenfeld residuals and visual inspection of scaled residual plots.

All analyses were conducted using R (R: A language and environment for statistical computing, R Foundation for Statistical Computing, Vienna, Austria, https://www.R-project.org, version 4.3.2), including the logistf package for Firth regression and pROC for ROC analysis. A two-sided p < 0.05 was considered statistically significant.

### Aim

The aim of this study was to validate commonly used RHF predictive risk models after durable LVAD implantation using a standardized INTERMACS-based RHF definition and to perform a secondary exploratory assessment of candidate multimodal predictors.

## Results

### Baseline characteristics and univariable associations

A total of 103 patients were included in the analysis, of whom 21 (20.4%) developed moderate–severe postoperative RHF. Baseline demographic, clinical, laboratory, and hemodynamic characteristics according to RHF status are summarized in Table 1.

**Table 1:** Baseline patient demographics, comorbidities and diagnostic findings.

Variables	No-mild RHF (n = 82)	Moderate-severe RHF (n = 21)	p value
**Basics**			
age, years	61.0 (54.2-66.8)	61.0 (50.0-68.0)	0.577
male gender	70 (85.4%)	17 (81.0%)	0.736
body mass index, kg/m^2^	27.5 (24.8-30.1)	27.7 (22.1-30.3)	1.000
body surface area, m^2^	2.0 (1.9-2.1)	2.1 (1.7-2.2)	0.809
**Comorbidities**			
diabetes mellitus	30 (36.6%)	7 (33.3%)	0.982
arterial hypertension	49 (59.8%)	10 (47.6%)	0.450
dyslipidemia	30 (36.6%)	6 (28.6%)	0.667
chronic obstructive pulmonary disease	14 (17.1%)	1 (4.8%)	0.727
renal failure	26 (31.7%)	4 (19%)	0.644
peripheral artery disease	6 (7.3%)	0 (0%)	0.803
atrial fibrillation	26 (31.7%)	5 (23.8%)	0.662
prior cardiac surgery	12 (14.6%)	8 (38.1%)	0.010
ICD^a^	36 (43.9%)	10 (47.6%)	0.306
**INTERMACS profile**			<0.001
I	8 (9.8%)	11 (52.4%)	
II	9 (11.0%)	4 (19.0%)	
III	28 (34.1%)	4 (19.0%)	
IV	37 (45.1%)	2 (9.5%)	
**Cardiac**			
cardiomyopathy			0.209
ischemic	60 (73.2%)	14 (66.7%)	
dilated	16 (19.5%)	4 (19.0%)	
others	6 (7.3%)	3 (14.3%)	
coronary artery disease			
including RCA stenosis	51 (62.2%)	14 (66.7%)	0.900
without RCA stenosis	17 (20.7%)	1 (4.8%)	0.162
previous percutaneous coronary intervention			0.464
including RCA	9 (11.0%)	1 (4.8%)	
without RCA	17 (20.7%)	5 (23.8%)	
**Prior organ support**			
intravenous inotropes	25 (30.5%)	11 (52.4%)	0.105
intravenous vasopressors	14 (17.1%)	10 (47.6%)	0.008
veno-arterial extracorporeal membrane oxygenation	6 (7.3%)	9 (42.9%)	<0.001
left-ventricular Impella®	8 (9.8%)	4 (19.0%)	0.259
mechanical ventilation	6 (7.3%)	9 (42.9%)	<0.001
dialysis	4 (4.9%)	3 (14.3%)	0.148
**Laboratory data**			
leukocytes,/µl	8.1 (6.6-10.1)	10.2 (6.8-11.3)	0.130
hemoglobin, g/dl	12.4 (10.0-13.8)	9.7 (8.6-12.6)	0.039
international normalized ratio	1.1 (1.0-1.2)	1.1 (1.1-1.3)	0.028
total bilirubin, mg/dl	0.5 (0.3-0.7)	1.0 (0.7-1.5)	<0.001
aspartate aminotransferase, U/l	27.0 (21.0-38.8)	33.0 (28.5-66.2)	0.018
alanine aminotransferase, U/l	29.0 (21.0-49.8)	35.0 (21.8-49.8)	0.685
troponin T, ng/l	35.0 (21.0-150.5)	73.0 (65.5-429.0)	0.121
creatine kinase, U/l	59.5 (36.2-117.5)	107.0 (52.0-214.0)	0.072
creatine kinase MB, U/l	14.0 (11.0-19.5)	15.5 (11.0-19.5)	0.843
creatinine, mg/dl	1.1 (0.9-1.5)	1.1 (0.9-1.6)	0.835
urea, mg/dl	45.0 (35.0-61.8)	59.0 (40.0-70.0)	0.092
NTproBNP, pg/ml	2921.0 (1598.5-5480.8)	3785.0 (2209.2-5448.5)	0.449
**Hemodynamics**			
heart rate, per min	74.0 (63.0-89.0)	83.0 (64.0-89.0)	0.284
systolic arterial pressure, mmHg	108.0 (101.0-122.0)	103.0 (93.0-121.0)	0.153
diastolic arterial pressure, mmHg	70.0 (61.0-79.0)	66.0 (59.0-73.0)	0.225
mean arterial pressure, mmHg	84.0 (74.0-91.0)	77.0 (73.0-84.0)	0.072
central venous pressure, mmHg	11.0 (8.0-15.0)	14.0 (7.8-15.2)	0.798
right atrial pressure, mmHg	10.0 (6.0-15.0)	14.0 (7.0-14.5)	0.479
systolic pulmonary artery (PA) pressure, mmHg	45 (33.0-59.8)	49.0 (35.0-57.5)	0.831
diastolic PA pressure, mmHg	22.0 (13.2-26.0)	28.0 (20.0-32.5)	0.034
mean PA pressure, mmHg	31.0 (21.0-37.5)	34.5 (25.5-39.0)	0.327
PA pulse pressure, mmHg	25.0 (18.0-32.8)	21.0 (12.5-30.5)	0.183
pulmonary capillary wedge pressure (PCWP), mmHg	21.0 (13.0-28.0)	22.5 (18.2-33.0)	0.231
transpulmonary gradient, mmHg	9.0 (5.5-12.0)	11.0 (7.0-14.0)	0.281
cardiac output, l/min	4.1 (3.6-4.7)	3.3 (2.9-4.1)	0.021
cardiac index, l/min/m^2^	2.0 (1.7-2.3)	1.8 (1.6-1.9)	0.021
stroke volume, ml	55.1 (45.6-67.1)	37.0 (32.2-53.9)	0.004
pulmonary vascular resistance, Wood units	2.2 (1.5-3.0)	3.9 (2.3-4.7)	0.023
systemic vascular resistance, dyne × sec/cm^5^	1440.0 (1173.0-1775.0)	1644.0 (1075.8-1912.2)	0.672
right atrial pressure/PCWP ratio	0.4 (0.3-0.6)	0.5 (0.3-0.7)	0.969
pulmonary artery pulsatility index	2.7 (1.9-4.5)	1.7 (1.0.-2.5)	0.057
right ventricular stroke work index, mmHg × l/m^2^	0.5 (0.3-0.7)	0.4 (0.2-0.7)	0.552
PA compliance, ml/mmHg	2.3 (1.8-3.7)	2.1 (1.2-2.7)	0.261
PA elastance, mmHg/ml	0.8 (0.6-1.1)	1.0 (0.9-1.8)	0.026
mixed venous oxygen saturation, %	59.5 (55.1-64.2)	66.7 (55.9-68.8)	0.538
central venous oxygen saturation, %	71.3 (64.2-73.0)	73.6 (68.7-78.2)	0.409
**Echocardiography**			
left-ventricular ejection fraction, %	23.0 (18.0-27.0)	18.0 (15.0-25.5)	0.206
left-ventricular enddiastolic diameter, mm	6.7 (6.2-7.2)	6.3 (5.7-7.1)	0.375
left-ventricular endsystolic diameter, mm	5.9 (5.4-6.5)	6.3 (5.6-7.3)	0.512
right-ventricular enddiastolic diameter, mm	4.0 (3.6-4.3)	4.8 (4.5-4.9)	0.050
tricuspid annular plane systolic excursion, mm	16.0 (11.0-20.0)	15.0 (15.0-16.0)	0.600
TAPSE/systolic PA pressure ratio	0.3 (0.2-0.5)	0.4 (0.3-0.4)	0.382
tricuspid annular systolic velocity, cm/sec	9.5 (7.0-11.8)	9.0 (7.0-9.5)	0.551
dilated inferior vena cava	8 (9.8%)	4 (19.0%)	0.422
right-ventricular dysfunction			0.252
none	38 (46.3%)	4 (19.0%)	
mild	19 (23.2%)	8 (38.1%)	
moderate	15 (18.3%)	5 (23.8%)	
severe	8 (9.8%)	3 (14.3%)	
aortic valve regurgitation			0.200
trivial or mild	27 (32.9%)	4 (19.0%)	
moderate	0 (0%)	1 (4.8%)	
severe	2 (2.4%)	0 (0%)	
mitral valve regurgitation			0.496
trivial or mild	44 (53.7%)	11 (52.4%)	
moderate	16 (19.5%)	7 (33.3%)	
severe	3 (3.7%)	1 (4.8%)	
tricuspid valve regurgitation			0.861
trivial or mild	41 (50.0%)	8 (38.1%)	
moderate	16 (19.5%)	4 (19.0%)	
severe	3 (3.7%)	1 (4.8%)	
left-ventricular aneurysm	7 (8.5%)	0 (0%)	0.337
left-ventricular thrombus	5 (6.1%)	4 (19.0%)	0.138

^a^including external wearable defibrillator and continuous monitoring after performed external defibrillation.

Abbreviations: ICD, internal cardioverter defibrillator; INTERMACS, Interagency Registry for Mechanically Assisted Circulatory Support; NTproBNP, N-terminal pro-B-type natiuretic peptide; PA, pulmonary artery; PCWP, pulmonary capillary wedge pressure; RCA, right coronary artery; RHF, right heart failure; TAPSE, tricuspid annular plane systolic excursion.

Patients who developed moderate–severe RHF presented with a substantially more critical INTERMACS profile than those without or with mild RHF (p < 0.001). They also had lower hemoglobin concentrations (9.7 vs. 12.4 g/dl, p = 0.039), higher international normalized ratio (1.1 (1.0–1.2) vs. 1.1 (1.1–1.3), p = 0.028), higher preoperative total bilirubin concentrations (1.0 vs. 0.5 mg/dl, p < 0.001), and higher aspartate aminotransferase levels (33.0 vs. 27.0 U/l, p = 0.018). Indices of impaired cardiac performance were more pronounced in the moderate–severe RHF group, including lower cardiac output (3.3 vs. 4.1 l/min, p = 0.021), lower cardiac index (1.8 vs. 2.0 l/min/m², p = 0.021), and lower stroke volume (37.0 vs. 55.1 ml, p = 0.004).

Pulmonary hemodynamics showed a more adverse profile in patients with moderate–severe RHF, who exhibited higher diastolic pulmonary artery pressure (28.0 vs. 22.0 mmHg, p = 0.034), higher pulmonary vascular resistance (3.9 vs. 2.2 Wood units, p = 0.023), and higher pulmonary artery elastance (1.0 vs. 0.8 mmHg/ml, p = 0.026). These findings suggest that markers of clinical acuity, hepatic congestion, reduced forward flow, and pulmonary vascular loading were associated with subsequent moderate–severe RHF in univariable analyses.

Data regarding the surgical approach are summarized in Table 2.

**Table 2:** Surgical procedure.

Variables	No-mild RHF (n = 82)	Moderate-severe RHF (n = 21)	p value
intention of treatment			0.007
bridge to recovery	2 (2.4%)	2 (9.5%)	
bridge to candidacy	32 (39.0%)	5 (23.8%)	
bridge to transplant	15 (18.3%)	2 (9.5%)	
bridge to decision	2 (2.4%)	4 (19.0%)	
destination therapy	31 (37.8%)	7 (33.3%)	
model			0.733
HeartMate 3^TM^	62 (75.6%)	17 (81.0%)	
HeartMate II®	17 (20.7%)	3 (14.3%)	
others	3 (3.6%)	1 (4.8%)	
surgical access			1.000
median sternotomy	68 (82.9%)	18 (85.7%)	
lateral thoracotomy	14 (17.1%)	3 (14.3%)	
cardiopulmonary bypass time, min	103.5 (81.0-134.0)	173.0 (133.0-192.0)	<0.001
cross clamp time, min	0.0 (0.0-35.5)	24.0 (12.0-36.0)	1.000
reperfusion time, min	0.0 (0.0-51.5)	49.0 (24.5-73.5)	0.653
concomitant coronary artery bypass grafting			0.768
without RCA	2 (2.4%)	0 (0.0%)	
with RCA	12 (14.6%)	3 (14.3%)	
concomitant valve procedure	5 (6.1%)	3 (14.3%)	0.355

Abbreviations: RCA, right coronary artery; RHF, right heart failure.

Most patients, 79 (76.7%), received a HeartMate 3™ VAD (Abbott Laboratories, Pleasanton, CA, USA), 20 (19.4%) were implanted a HeartMate II® (Thoratec Corporation, Pleasanton, CA, USA; now Abbott Laboratories, Abbott Park, IL, USA), a BerlinHeart Excor® (Berlin Heart GmbH, Berlin, Germany; now Getinge Group, Gothenburg, Sweden) was implanted in two (1,9%), and each one (1.0%) received a HeartWare HVAD® (HeartWare Inc., Framingham, MA, USA; now Medtronic, Minneapolis, MN, USA) and a BerlinHeart Incor® (Berlin Heart GmbH, Berlin, Germany; now Getinge Group, Gothenburg, Sweden). Notably, cardiopulmonary bypass time was significantly longer in the moderate-severe RHF group (p < 0.001).

Postoperative outcomes (Table 3) likewise differed markedly between groups. Patients with moderate–severe RHF required longer periods of intravenous inotropic support (p < 0.001), intravenous vasodilator therapy (p = 0.008), and inhaled nitric oxide therapy (p = 0.004). Secondary right-ventricular mechanical circulatory support was substantially more common in the moderate–severe RHF group (76.2% vs. 0.0%, p < 0.001). Moderate–severe RHF was also associated with more frequent reoperation for bleeding (61.9% vs. 20.7%, p < 0.001), respiratory failure (76.2% vs. 22.0%, p < 0.001), renal failure requiring dialysis (90.5% vs. 29.3%, p < 0.001), and liver failure requiring extracorporeal detoxification (38.1% vs. 3.7%, p < 0.001). Hospital stay was significantly longer in patients with moderate–severe RHF (59.0 vs. 23.0 days, p = 0.005), whereas the difference in intensive care unit stay did not reach statistical significance (25.5 vs. 9.5 days, p = 0.178).

**Table 3:** Patient outcomes and adverse events.

Variables	No-mild RHF (n = 82)	Moderate-severe RHF (n = 21)	p value
**Right ventricular failure**			
extracorporeal membrane oxygenation			<0.001
veno-arterial	1 (1.2%)	1 (4.8%)	
veno-pulmonalarterial	0 (0.0%)	6 (28.6%)	
right-ventricular Impella®	0 (0.0%)	1 (4.8%)	0.204
thorax apertus	1 (1.2%)	3 (14.3%)	0.026
duration of intravenous inotropes			<0.001
<7 days	48 (58.5%)	8 (38.1%)	
7-14 days	23 (28.0%)	5 (23.8%)	
>14 days	2 (2.4%)	7 (33.3%)	
duration of intravenous vasodilators			0.008
<7 days	0 (0.0%)	3 (14.3%)	
7-14 days	0 (0.0%)	0 (0.0%)	
>14days	0 (0.0%)	0 (0.0%)	
duration of inhaled nitric oxide			0.004
<7 days	41 (50.0%)	17 (81.0%)	
7-14 days	2 (2.4%)	1 (4.8%)	
>14 days	0 (0.0%)	1 (4.8%)	
secondary right ventricular mechanical circulatory support	0 (0.0%)	16 (76.2%)	<0.001
**other severe organ dysfunction**			
bleeding, requiring reoperation	17 (20.7%)	13 (61.9%)	<0.001
tamponade	14 (17.1%)	5 (23.8%)	0.531
wound infection	3 (3.7%)	0 (0.0%)	1.000
severe infection/sepsis	54 (65.9%)	13 (61.9%)	0.935
new onset ventricular arrhythmia	11 (13.4%)	6 (28.6%)	0.108
respiratory failure (reintubation/tracheostomy)	18 (22.0%)	16 (76.2%)	<0.001
renal failure, requiring dialysis	24 (29.3%)	19 (90.5%)	<0.001
liver failure, requiring extracorporeal detoxification	3 (3.7%)	8 (38.1%)	<0.001
**discharge and survival**			
intensive care unit stay, days	9.5 (5.0-25.8)	25.5 (7.0-38.0)	0.178
hospital stay, days	23.0 (17.0-35.5)	59.0 (44.0-71.0)	0.005
in-hospital death due to RHF	1 (1.2%)	11 (52.4%)	<0.001
in-hospital death due to other cause	7 (8.5%)	4 (19.0%)	0.229
time till death of any cause, days	749.5 (124.8-1802.5)	18.0 (7.0-29.0)	<0.001

Abbreviations: RHF, right heart failure.

All variables with a significance level of p < 0.05 in the univariable screening were included in the complete multivariable model, which is provided in S2 Table.

### Multivariable modelling

Because of the limited number of moderate–severe RHF events, Firth-penalized logistic regression was applied to obtain a stable, parsimonious exploratory model. Five variables were retained based on clinical relevance, avoidance of redundancy among correlated physiological domains, and statistical stability: INTERMACS profile, total bilirubin, aspartate aminotransferase, diastolic PA pressure, and pulmonary vascular resistance.

In the Firth-penalized model, only INTERMACS profile remained independently associated with moderate–severe RHF (adjusted OR 0.40, 95% CI 0.18–0.79; p = 0.008). Total bilirubin, aspartate aminotransferase, diastolic PA pressure, and pulmonary vascular resistance showed no independent statistically significant association after adjustment. Detailed results of the exploratory reduced model are shown in Table 4.

**Table 4:** Exploratory multivariable Firth logistic regression model for predicting moderate–severe right heart failure.

Variables	Adjusted odds ratio	95% confidence interval	p value
INTERMACS profile	0.40	0.18-0.79	0.008
total bilirubin	1.41	0.50-4.10	0.503
diastolic pulmonary artery pressure	1.05	0.97-1.16	0.215
aspartate aminotransferase	1.01	0.98-1.03	0.664
pulmonary vascular resistance	0.99	0.63-1.44	0.975

Abbreviations: INTERMACS, Interagency Registry for Mechanically Assisted Circulatory Support.

### Model performance compared with existing RHF scores

The discriminative ability of established RHF prediction scores is summarized in Table 5. Across all tested established scores, AUC values ranged from 0.394 (Utah score) to 0.729 (postoperative EUROMACS score). Because the postoperative EUROMACS score includes cardiopulmonary bypass time, its performance should be interpreted as early perioperative/dynamic discrimination rather than as purely preoperative prediction. INTERMACS profile alone demonstrated robust apparent discriminative performance with an AUC of 0.803.

**Table 5:** Discrimination of right heart failure prediction models.

Model/Score	AUC	95% confidence interval
preoperative EUROMACS	0.481	0.314-0.645
postoperative EUROMACS	0.729	0.591-0.844
CRITT	0.587	0.462-0.704
Michigan	0.699	0.583-0.813
BiVAD score	0.487	0.346-0.628
Utah score	0.394	0.278-0.514
modified Utah score	0.444	0.314-0.581
INTERMACS profile	0.803	0.678-0.900
Exploratory five-variable Firth model (apparent performance)	0.854	0.741-0.939

Abbreviations: AUC, area under the curve; BiVAD, biventricular assist device; CRITT, central venous pressure, right-ventricular dysfunction, intubation, tricuspid valve regurgitation, tachycardia; EUROMACS, European Registry for Patients with Mechanical Circulatory Support; INTERMACS, Interagency Registry for Mechanically Assisted Circulatory Support.

The exploratory five-variable Firth model achieved the highest apparent discrimination (AUC 0.854, 95% CI 0.741–0.939). Sensitivity and specificity metrics for each score and for the exploratory Firth model are reported in S3 Table. Because this model was derived and evaluated in the same cohort, bootstrap internal validation was performed to quantify optimism (Table 6).

**Table 6:** Bootstrap internal validation of the exploratory five-variable Firth model for prediction of moderate–severe right heart failure.

Performance measure	Apparent estimate	Mean bootstrap optimism	Optimism-corrected estimate
AUC	0.854	0.081	0.773
Calibration intercept	−0.091	0.024	−0.115
Calibration slope	1.062	0.476	0.586
Brier score	0.114	0.035	0.149

Abbreviations: AUC, area under the receiver-operating characteristic curve. Bootstrap internal validation was performed using 2,000 bootstrap resamples in the complete-case dataset used for the exploratory five-variable Firth model (n = 65; moderate–severe RHF events, n = 12). The fixed final model included INTERMACS profile, total bilirubin, aspartate aminotransferase, diastolic pulmonary artery pressure, and pulmonary vascular resistance. Optimism-corrected estimates were calculated by subtracting the mean bootstrap optimism from the apparent estimate. For the Brier score, lower values indicate better overall prediction accuracy. The optimism-corrected calibration slope below 1 indicates relevant model optimism and potential overfitting.

After bootstrap correction, the AUC decreased from 0.854 to 0.773, and the optimism-corrected calibration slope was 0.586. These findings indicate relevant optimism and support cautious interpretation of the model as exploratory and hypothesis-generating rather than as a validated prediction tool. A calibration plot is provided in supplementary figure S1 Fig.

**Fig 1:**
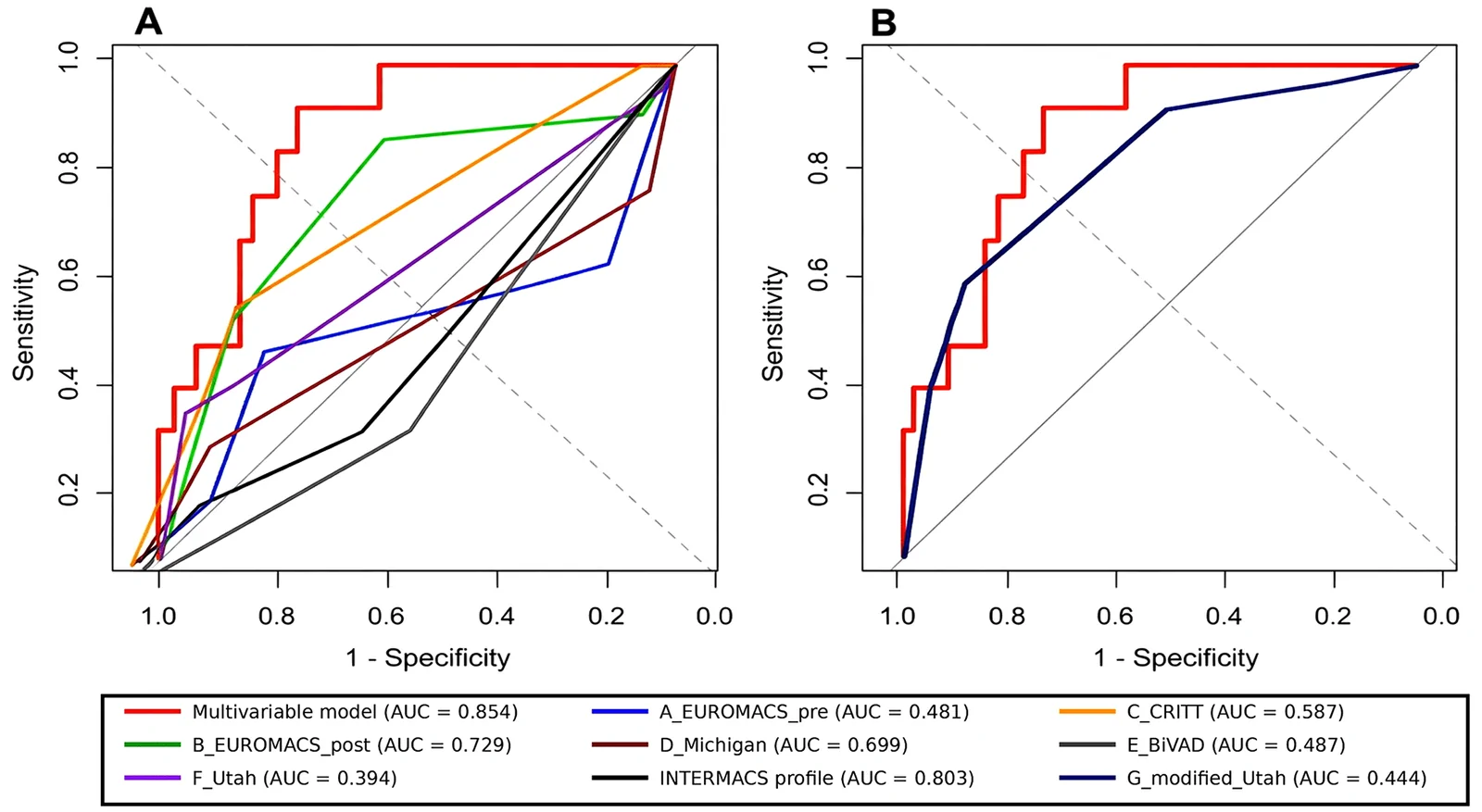
Receiver operating characteristic curves of right heart failure prediction models. A: Established right heart failure scores versus the exploratory five-variable Firth model. B: INTERMACS profile versus the exploratory five-variable Firth model. Curves show apparent discrimination before bootstrap optimism correction.

Corresponding apparent ROC curves are illustrated in Fig 1, comparing (Panel A) the exploratory Firth model with established RHF scores and (Panel B) the exploratory Firth model with INTERMACS profile alone.

### Survival outcomes

**Fig 2:**
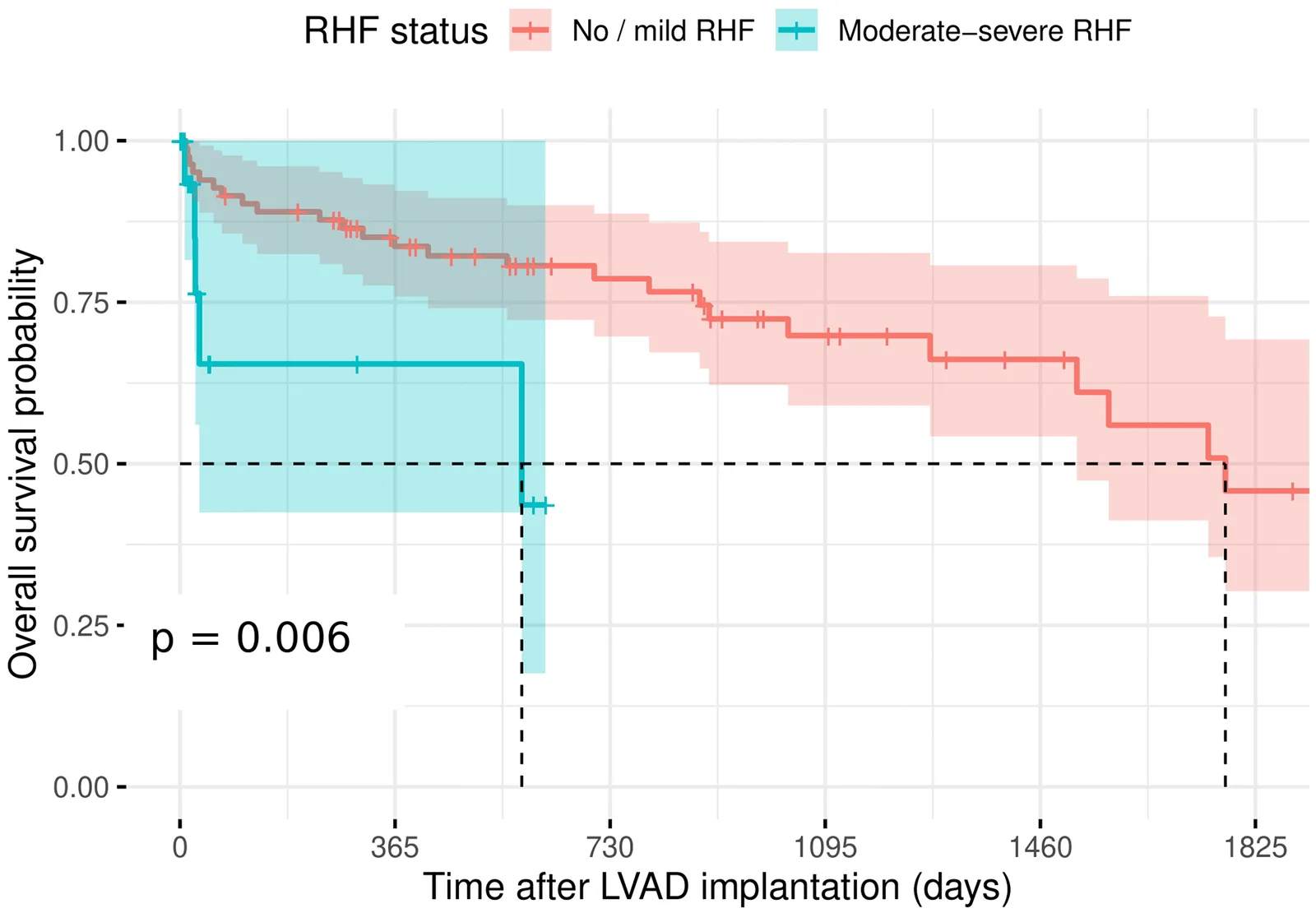
Kaplan–Meier curves of survival after durable left ventricular assist device implantation stratified by right heart failure severity. Red: no or mild right heart failure. Green: moderate–severe right heart failure.

Overall survival was significantly reduced among patients who developed moderate–severe RHF. Kaplan–Meier curves demonstrated an early divergence between groups (Fig 2). Thirty-day survival was 98.8% in the no-mild RHF group compared with 76.2% among moderate-severe RHF patients. One-year survival was 79.3% versus 57.1%, respectively. The log-rank test confirmed a statistically significant difference (p = 0.006).

In unadjusted Cox regression, the presence of moderate-severe RHF was associated with a significantly increased risk of mortality (HR 3.83, 95% CI 1.36–10.80; p = 0.011).

## Discussion

Perioperative RHF remains a serious complication following the implantation of durable ventricular assist devices. Given the complex nature of RHF, effective preoperative risk stratification is challenging but essential for improving patient outcomes and determining the appropriate timing and use of advanced medical or mechanical biventricular support.

Our study externally validated established RHF predictive risk models in a consecutive single-center cohort of patients undergoing primary durable LVAD implantation. One of our major findings was the limited-to-moderate discrimination of existing RHF risk models when applied with a contemporary INTERMACS-based endpoint definition. The postoperative EUROMACS-RHF score demonstrated the highest discriminatory ability among established models, with an AUC of 0.729. However, this score includes cardiopulmonary bypass time and therefore represents an early perioperative/dynamic risk marker rather than a purely preoperative prediction model. This temporal difference must be considered when comparing it with preoperative scores. Overall, the modest performance of established scores aligns with prior work suggesting limited transportability across heterogeneous LVAD populations, institutional treatment protocols, and RHF definitions [3,20–22].

Interestingly, the preoperative EUROMACS, CRITT, BiVAD, and Utah RVF scores did not show strong discriminatory performance in our real-world cohort (S4 Table), consistent with reports suggesting that these models have not been consistently validated in heterogeneous populations [23–25]. The Utah score performed poorly with an AUC of 0.394. Because intra-aortic balloon pump support is now rarely used as temporary MCS in our center, we calculated a modified Utah score by grouping extracorporeal membrane oxygenation and/or Impella® with intra-aortic balloon pump support. This modification increased the AUC only slightly to 0.444 and should be interpreted only as an exploratory sensitivity analysis because it has not been externally validated.

Our study suggests that comprehensive, multimodal preoperative assessment remains important for RHF risk stratification. Clinical acuity reflected by INTERMACS profile, markers of hepatic congestion, and pulmonary hemodynamic parameters were associated with moderate–severe RHF in univariable analyses. In the exploratory Firth-penalized model, however, only INTERMACS profile remained independently associated with RHF. INTERMACS profile alone also showed robust apparent discrimination (AUC 0.803), highlighting the importance of global clinical status and preoperative shock severity.

The exploratory five-variable Firth model showed the highest apparent discrimination (AUC 0.854), but internal validation demonstrated relevant optimism. After 2,000 bootstrap resamples, the optimism-corrected AUC decreased to 0.773 and the optimism-corrected calibration slope was 0.586. These findings indicate relevant optimism and potential overfitting, as reflected by the optimism-corrected calibration slope of 0.586. The model should therefore not be interpreted as a validated clinical score. Instead, it should be viewed as a hypothesis-generating signal suggesting that a combination of preoperative clinical status, hepatic laboratory markers, and pulmonary hemodynamics may be useful for future prediction models developed in larger multicenter cohorts.

A particularly interesting aspect of our study is that several commonly used hemodynamic parameters, such as right ventricular stroke work index, PCWP, PA compliance, and PA pulsatility index, did not emerge as independent predictors in the multivariable model. Several explanations are possible. First, invasive hemodynamic measurements in advanced heart failure are highly dependent on loading conditions, temporary MCS, ventilatory support, inotropes, vasopressors, and volume status at the time of assessment. Second, static resting measurements may not capture right ventricular reserve or the dynamic response to LVAD-induced preload shifts. Third, missingness and the limited number of events reduced statistical power and increased uncertainty. These limitations support the need for dynamic, multimodal, and externally validated approaches rather than reliance on individual hemodynamic thresholds.

Cardiopulmonary bypass time was significantly prolonged in our RHF group, reinforcing previous studies that suggest prolonged bypass times can contribute to postoperative RHF by causing hemodynamic instability and right ventricular dysfunction [6]. While device technology has improved dramatically, the importance of surgical precision and perioperative management should not be underestimated in predicting the risk for RHF. As LVAD implantation procedures continue to evolve, it will become crucial to refine intraoperative risk awareness to account for surgical factors that influence postoperative outcomes.

Several limitations of this study must be acknowledged. First, this was a single-center retrospective analysis, which introduces potential selection bias and limits generalizability to other LVAD populations and perioperative treatment protocols. Second, the number of moderate–severe RHF events was limited (n = 21), and the exploratory Firth model was fitted in a restricted complete-case dataset of 65 patients with only 12 events. Third, missing values were not imputed, which may have introduced additional selection bias in complete-case analyses. Fourth, although Firth regression and bootstrap optimism correction were used to reduce small-sample bias and quantify overfitting, the exploratory model was derived and internally validated in the same cohort and lacks external validation. Fifth, the postoperative EUROMACS score includes cardiopulmonary bypass time and is therefore not directly comparable to purely preoperative risk scores; it may be advantaged by perioperative information and is susceptible to temporal bias. Finally, reliance on existing RHF definitions and retrospective endpoint adjudication may introduce variability compared with prospective adjudication.

Future research should focus on multicenter validation of the identified candidate predictors and on prospective development of RHF prediction models using standardized endpoint definitions. Larger datasets would allow robust internal-external validation, calibration assessment, and evaluation of whether dynamic perioperative variables, advanced imaging parameters, biomarkers, or explainable machine-learning approaches can improve prediction beyond clinical status alone. Any machine-learning approach should be evaluated with transparent reporting, calibration, and external validation before clinical implementation [26,27].

## Conclusion

In conclusion, most established RHF risk scores showed limited-to-moderate discrimination for predicting moderate–severe RHF after durable LVAD implantation in this single-center cohort. Postoperative EUROMACS demonstrated the highest AUC among established scores but should be considered an early perioperative/dynamic marker rather than a purely preoperative prediction tool. The exploratory five-variable Firth model suggested that clinical acuity, hepatic laboratory markers, and pulmonary hemodynamics may provide complementary information; however, bootstrap validation showed relevant optimism. Therefore, this model should be interpreted as hypothesis-generating and requires validation in larger independent multicenter cohorts before any clinical use can be considered. Future risk stratification should prioritize externally validated models developed in larger multicenter cohorts.

## Supporting information

S1 Fig Calibration plot of the exploratory five-variable Firth model for prediction of moderate–severe right heart failure. Predicted probabilities were generated from the final fixed five-variable Firth-penalized logistic regression model in the complete-case dataset (n = 65; moderate–severe RHF events, n = 12). The diagonal reference line represents perfect calibration. Points represent grouped observed event rates across predicted-risk strata. Because of the limited number of events and the single-center design, this calibration analysis should be interpreted as exploratory.
(TIF)

S1 Table Parameters and calculation of established right heart failure predictive risk models. Abbreviations: ACE, angiotensin converting enzyme; ARB, angiotensin receptor blocker; BiVAD, biventricular assist device; CRITT, central venous pressure, right-ventricular dysfunction, intubation, tricuspid valve regurgitation, tachycardia; EUROMACS, European Registry for Patients with Mechanical Circulatory Support; INTERMACS, Interagency Registry for Mechanically Assisted Circulatory Support; LVAD, left-ventricular assist device; PCWP, pulmonary capillary wedge pressure; RHF, right heart failure; RVAD, right ventricular assist device; RVF, right-ventricular failure; RVSWI, right ventricular stroke work index.
(DOCX)

S2 Table Full multivariable logistic regression model. This table shows the exploratory full multivariable logistic regression model including all variables with p < 0.05 in univariate analysis. Abbreviations: CI, confidence interval; INTERMACS, Interagency Registry for Mechanically Assisted Circulatory Support; OR, odds ratio.
(DOCX)

S3 Table Sensitivity and specificity of right heart failure predictive risk models. Abbreviations: BiVAD, biventricular assist device; EUROMACS, European Registry for Patients with Mechanical Circulatory Support; INTERMACS, Interagency Registry for Mechanically Assisted Circulatory Support.
(DOCX)

S4 Table Univariate analysis of established right heart failure predictive risk models. P values were calculated using the Mann-Whitney U test for the coded ordinal score variable, consistent with the ROC analyses. Patients without an evaluable score were retained in the descriptive table as not evaluable. Abbreviations: BiVAD, biventricular assist device; EUROMACS, European Registry for Patients with Mechanical Circulatory Support; LVAD, left-ventricular assist device; RHF, right heart failure; RVAD, right ventricular assist device; RVF, right-ventricular failure.
(DOCX)
