# Supplementary material for: Predicting the right! Validation of right heart failure predictive risk models after primary durable ventricular assist device implantation
Source: PLoS One. 2026 Sep 9;21(9):e0357025. doi: 10.1371/journal.pone.0357025 (PMC13557412; doi:10.1371/journal.pone.0357025)
Supplement: Supplementary file 1 [file pone.0357025.s002.docx]

| Predictive risk model | score components | points | results |
| --- | --- | --- | --- |
| preoperative EUROMACS-RHF risk score | right atrial pressure/PCWP ratio >.54  hemoglobin ≤ 10 g/dl  ≥ 3 intravenous inotropes  INTERMACS I-III  sever right-ventricular dysfunction | 2  1  2.5  2  2 | 0 – 2 points:  low risk for RHF  2.5 – 4 points:  medium risk for RHF  > 4 points:  high risk for RHF |
| postoperative EUROMACS-RHF risk score | right atrial pressure/PCWP ratio >.54  hemoglobin ≤ 10g/dl  ≥ 3 intravenous inotropes  INTERMACS I-III  sever right-ventricular dysfunction  cardiopulmonary bypass time > 100 min | 1  1.5  2  2  1  1 | as above |
| Michigan RVF risk score | intravenous vasopressors  aspartate aminotransferase ≥ 80 U/l  total bilirubin ≥ 2.0 mg/dl  creatinine ≥ 2.3 mg/dl | 4  2  2.5  3 | ≤ 3 points:  low risk for RHF  4 – 5 points:  medium risk for RHF  ≥ 5.5 points:  high risk for RHF |
| CRITT score | **C**entral venous pressure > 15 mmHg  sever **r**ight-ventricular dysfunction  **i**ntubation  severe **t**ricuspid valve regurgitation  **t**achycardia > 100/min | 1 for each | 0 – 1 points:  isolated LVAD  2 – 3 points:  LVAD with advanced medication or temporary RVAD  4 – 5 points:  BiVAD recommended |
| BiVAD score | cardiac index ≤ 2.2 l/min/m^2^  RVSWI ≤ .25 mmHg × l/m^2^  severe right ventricular dysfunction  creatinine ≥ 1.9 mg/dl  prior cardiac surgery  systolic blood pressure ≤ 96 mmHg | 18  18  16  17  16  13 | 0 – 49 points:  isolated LVAD  50 – 98 points:  BiVAD necessary |
| Utah RVF risk score | destination therapy  on intra-aortic balloon pump support  pulmonary vascular resistance  ≤ 1.7 Wood units  1.8 – 2.7 Wood units  2.8 – 4.2 Wood units  ≥ 4.3 Wood units  on intravenous inotropes  obesity  on ACE inhibitor or ARB  on β blocker | 3.5  4  1  2  3  4  2.5  2  2.5  2 | ≤ 5 points:  low risk for RHF  5.5 – 8.0 points:  medium risk for RHF  8.5 – 12 points:  increased risk for RHF  ≥ 12.5 points:  high risk for RHF |
