# Supplementary material for: Predicting the right! Validation of right heart failure predictive risk models after primary durable ventricular assist device implantation
Source: PLoS One. 2026 Sep 9;21(9):e0357025. doi: 10.1371/journal.pone.0357025 (PMC13557412; doi:10.1371/journal.pone.0357025)
Supplement: Supplementary file 2 [file pone.0357025.s003.docx]

| Variables | Adjusted OR (95% CI) | p value |
| --- | --- | --- |
| INTERMACS profile | 0.28 (0.08–1.04) | 0.057 |
| hemoglobin | 1.10 (0.68–1.79) | 0.694 |
| international normalized ratio | 0.10 (0.00–12.25) | 0.346 |
| total bilirubin | 2.00 (0.44–8.99) | 0.367 |
| aspartate aminotransferase | 1.01 (0.98–1.04) | 0.590 |
| diastolic pulmonary artery pressure | 1.15 (0.94–1.42) | 0.179 |
| pulmonary vascular resistance | 0.95 (0.54–1.66) | 0.851 |
| pulmonary arterial elastance | 0.12 (0.00–8.82) | 0.336 |
| right atrial pressure | 0.98 (0.76–1.27) | 0.884 |
| stroke volume index | 0.00 (0.00–1.59×10¹⁶) | 0.141 |
| number of vasopressors | 0.41 (0.06–2.96) | 0.374 |
| cardiopulmonary bypass time | 1.01 (0.99–1.02) | 0.320 |
