# Supplementary material for: Predicting the right! Validation of right heart failure predictive risk models after primary durable ventricular assist device implantation
Source: PLoS One. 2026 Sep 9;21(9):e0357025. doi: 10.1371/journal.pone.0357025 (PMC13557412; doi:10.1371/journal.pone.0357025)
Supplement: Supplementary file 3 [file pone.0357025.s004.docx]

| Score/model | sensitivity | specificity |
| --- | --- | --- |
| preoperative EUROMACS | 0.38 | 0.57 |
| postoperative EUROMACS | 0.52 | 0.74 |
| CRITT | 0.43 | 0.65 |
| Michigan | 0.48 | 0.70 |
| BiVAD score | 0.29 | 0.61 |
| Utah score | 0.22 | 0.68 |
| Modified Utah | 0.27 | 0.66 |
| INTERMACS profile | 0.67 | 0.78 |
| 5-variable Firth model | 0.81 | 0.83 |
