# Supplementary material for: Predicting the right! Validation of right heart failure predictive risk models after primary durable ventricular assist device implantation
Source: PLoS One. 2026 Sep 9;21(9):e0357025. doi: 10.1371/journal.pone.0357025 (PMC13557412; doi:10.1371/journal.pone.0357025)
Supplement: Supplementary file 4 [file pone.0357025.s005.docx]

| RHF predictive risk model | No-mild RHF  (n = 82) | Moderate-severe RHF  (n = 21) | p value |
| --- | --- | --- | --- |
| preoperative EUROMACS-RHF risk score |  |  | **0.772** |
| low risk | 53 (64.6%) | 4 (19.0%) |  |
| moderate risk | 9 (11.0%) | 7 (33.3%) |  |
| high risk | 9 (11.0%) | 2 (9.5%) |  |
| not evaluable | 11 (13.4%) | 8 (38.1%) |  |
| postoperative EUROMACS-RHF risk score |  |  | **<0.001** |
| low risk | 41 (50.0%) | 1 (4.8%) |  |
| moderate risk | 22 (26.8%) | 7 (33.3%) |  |
| high risk | 14 (17.1%) | 11 (52.4%) |  |
| not evaluable | 5 (6.1%) | 2 (9.5%) |  |
| CRITT score |  |  | **0.080** |
| isolated LVAD | 69 (84.1%) | 13 (61.9%) |  |
| LVAD with advanced medication or temporary RVAD | 11 (13.4%) | 7 (33.3%) |  |
| implantation BiVAD necessary | 0 (0.0%) | 0 (0.0%) |  |
| not evaluable | 2 (2.4%) | 1 (4.8%) |  |
| Michigan RVF risk score |  |  | **<0.001** |
| low risk | 63 (76.8%) | 10 (47.6%) |  |
| moderate risk | 9 (11.0%) | 5 (23.8%) |  |
| high risk | 5 (6.1%) | 6 (28.6%) |  |
| not evaluable | 5 (6.1%) | 0 (0.0%) |  |
| BiVAD score |  |  | **0.764** |
| isolated LVAD | 76 (92.7%) | 12 (57.1%) |  |
| implantation BiVAD necessary | 2 (2.4%) | 4 (19.0%) |  |
| not evaluable | 4 (4.9%) | 5 (23.8%) |  |
| Utah RVF risk score |  |  | **0.126** |
| low risk | 28 (34.1%) | 10 (47.6%) |  |
| moderate risk | 25 (30.5%) | 4 (19.0%) |  |
| increased risk | 5 (6.1%) | 0 (0.0%) |  |
| high risk | 0 (0.0%) | 0 (0.0%) |  |
| not evaluable | 24 (29.3%) | 7 (33.3%) |  |
| modified Utah RVF risk score |  |  | **0.407** |
| low risk | 30 (36.6%) | 10 (47.6%) |  |
| moderate risk | 23 (28.0%) | 2 (9.5%) |  |
| increased risk | 7 (8.5%) | 3 (14.3%) |  |
| high risk | 0 (0.0%) | 0 (0.0%) |  |
| not evaluable | 22 (26.8%) | 6 (28.6%) |  |
