# Supplementary material for: Predicting the right! Validation of right heart failure predictive risk models after primary durable ventricular assist device implantation
Source: PLoS One. 2026 Sep 9;21(9):e0357025. doi: 10.1371/journal.pone.0357025 (PMC13557412; doi:10.1371/journal.pone.0357025)
Supplement: Supplementary file 5 [file pone.0357025.s007.docx]

**RESPONSE TO REVIEWERS**

**Manuscript ID:** PONE-D-26-07516

**Title:** Predicting the right! Validation of right heart failure predictive risk models after primary durable ventricular assist device implantation

**Journal:** PLOS ONE

Dear Academic Editor and Reviewers,

We thank the Academic Editor and both reviewers for their careful evaluation of our manuscript and for the constructive comments. We have revised the manuscript substantially to clarify the methodological framework, moderate the interpretation of the exploratory Firth model, add bootstrap internal validation and calibration analyses, clarify the timing and role of the postoperative EUROMACS score, and improve language, formatting, and consistency throughout the manuscript. We also completed S4 Table in the Supporting information. All changes are highlighted in the marked-up manuscript and incorporated in the clean revised manuscript. Page and line numbers below refer to the clean revised manuscript unless otherwise stated.

**Academic Editor Comments**

**Editor comment 1: Firth model development, internal validation, and interpretation**

We agree with the Academic Editor. We revised the manuscript to clearly distinguish the primary external validation of established RHF scores from the secondary exploratory analysis of the five-variable Firth model. We added bootstrap internal validation using 2,000 bootstrap resamples and now report optimism-corrected discrimination and calibration metrics. The apparent AUC of the exploratory model was 0.854 and decreased to an optimism-corrected AUC of **0.773**; the optimism-corrected calibration slope was **0.586**, indicating relevant optimism and potential overfitting. We therefore no longer describe the model as superior or clinically ready and consistently present it as exploratory and hypothesis-generating.

**Changes made:**

- Revised Abstract (Results and Conclusions)
- Added bootstrap description in **Methods – Statistical analysis** (new paragraph after the description of the Firth model)
- Added new **Table 6** (Bootstrap internal validation) placed directly after Table 5 in the Results section
- Added reference to new **S1 Fig** (Calibration plot) after Figure 2
- Revised the paragraph discussing the Firth model in the **Discussion**
- Strengthened the **Limitations** section

**Location in revised files:** Abstract, p. 2, lines 18–51; Methods–Statistical analysis, pp. 7-10, lines 166–228; Results (Table 5–6 and interpretation), pp. 16-17, lines 316–349; Discussion, pp. 18–19, lines 370–405; Limitations, pp. 20-21, lines 424–444; Conclusion, p. 21, lines 447–457; Supporting information, S1 Fig., S2-S4 Table.

**Editor comment 2: Timing and rationale of postoperative EUROMACS**

We agree that the postoperative EUROMACS score is conceptually different from purely preoperative scores because it includes cardiopulmonary bypass time, which is only available after LVAD implantation. We clarified that postoperative EUROMACS was analyzed separately as an early perioperative/dynamic risk marker and should not be interpreted as a purely preoperative prediction model. We also added cautious language about temporal bias and its limited comparability with preoperative scores.

**Changes made:**

- Revised **Methods – Right heart failure predictive risk models**
- Updated **Results – Model performance** section
- Added explanation in **Discussion** and **Limitations**

**Location in revised files:** Abstract, pp. 2-3, lines 28–36 and 46–51; Methods–Right heart failure predictive risk models, pp. 6-7, lines 147–165; Results–Model performance, pp. 15-16, lines 308–315; Discussion, p. 18, lines 366–380; Limitations, p. 20, lines 424–437; Conclusion, p. 21, lines 446–457.

**Editor comment 3: Data availability contact point**

We revised the Data Availability Statement to provide a non-author institutional contact. The revised statement directs data requests to the Ethics Committee of the Medical Faculty of RWTH Aachen University and includes institutional contact information and the ethics approval number.

**Changes made:**

- Updated **Data Availability Statement**

**Location in revised files:** Data Availability Statement

**Reviewer #1 Comments**

**Reviewer #1, minor comment 1: Language and formatting**

We thank the reviewer. We revised the manuscript throughout for clarity, corrected typographical and spacing issues, and standardized terminology. We use RHF for right heart failure, RVF only when referring to the original names of published scores, and LVAD consistently. We also changed “5-variables” to “five-variable” and standardized “moderate–severe RHF”.

**Changes made:**

- Language and terminology edits throughout the manuscript
- Standardized figure and Supporting information captions

**Location in revised files:** Abstract, p. 2, lines 18–51; Methods–Right heart failure predictive risk models, pp. 6-7, lines 147–165; Figure 1 caption, p. 17, lines 345–350; Figure 2 caption, p. 17, lines 357–359; Supporting information captions.

**Reviewer #1, minor comment 2: AUC confidence intervals and transition to exploratory model**

We agree. Table 5 already reports AUC values with 95% confidence intervals. We revised the Results and Discussion to better separate validation of established scores from the secondary exploratory Firth model. We added **Table 6** with bootstrap internal validation and calibration metrics, which provides a clearer transition from apparent to optimism-corrected performance. We also completed **S4 Table** in the Supporting information.

**Changes made:**

- Revised Results and Discussion for clearer separation of topics
- Added new **Table 6** after Table 5
- Completed **S4 Table** in the Supporting information

**Location in revised files:** Results (Table 5–6), p. 16, lines 316–329; Discussion, pp. 18–19, lines 365–405; Supporting information, completed S4 Table.

**Reviewer #1, minor comment 3: Modified Utah score**

We agree. We clarified that the original Utah score includes intra-aortic balloon pump support, whereas ECMO and Impella® are now more commonly used as temporary MCS in our center. We explicitly state that the modified Utah score has not been externally validated and was analyzed only as an exploratory sensitivity analysis.

**Changes made:**

- Added clarification in **Methods – Right heart failure predictive risk models**
- Updated **Discussion**

**Location in revised files:** Methods, p. 7, lines 160–165; Discussion, pp. 18-19, lines 381–389; Supporting information, S4 Table.

**Reviewer #1, minor comment 4: Statistical methods**

We expanded the statistical methods section. We explain that standard logistic regression showed separation tendencies and convergence warnings due to the small event-to-variable ratio. Firth bias-reduced penalized logistic regression was therefore used to reduce small-sample bias and improve estimate stability. We also clarified how variables were selected for the final exploratory model and explicitly state that the model was not intended to represent a validated clinical score.

**Changes made:**

- Expanded justification for Firth regression in **Methods – Statistical analysis**
- Clarified variable selection process

**Location in revised files:** Methods–Statistical analysis, pp. 7-9, lines 166–206; Results–Multivariable modelling, p. 15, lines 291–301.

**Reviewer #1, minor comment 5: Hemodynamic parameters**

We agree and expanded the Discussion. We now explain that invasive hemodynamic measurements in advanced heart failure are affected by loading conditions, temporary MCS, ventilatory support, vasoactive medication, and volume status. We also note that static resting measurements may not capture right ventricular reserve or the dynamic response to LVAD-induced preload shifts, and that missingness and limited event number reduced statistical power.

**Changes made:**

- Expanded discussion of hemodynamic parameters in the **Discussion** section

**Location in revised files:** Discussion, pp. 19-20, lines 406–416.

**Reviewer #1, minor comment 6: Limitations**

We agree. We revised the Limitations section to explicitly mention single-center selection bias, limited generalizability, missing data, complete-case analysis (n = 65 with only 12 events), absence of imputation, and lack of external validation of the exploratory model.

**Changes made:**

- Strengthened **Limitations** section with more specific points

**Location in revised files:** Methods–Statistical analysis, p. 7, lines 169–173; Limitations, p. 20, lines 424–437.

**Reviewer #1, minor comment 7: Future directions**

We revised the future-directions paragraph to make it more specific. We now state that larger multicenter datasets are required for robust internal-external validation, calibration assessment, and evaluation of dynamic perioperative variables, advanced imaging, biomarkers, or explainable machine-learning approaches. We emphasize that such approaches require transparent reporting, calibration, and external validation before clinical implementation.

**Changes made:**

- Made future research paragraph more specific and actionable

**Location in revised files:** Future research paragraph, pp. 20-21, lines 438–444.

**Reviewer #2 Comments**

**Reviewer #2, major comment 1: Overfitting and self-derived model**

We fully agree with this concern. We performed bootstrap internal validation using **2,000 resamples**. The exploratory Firth model was fitted in the complete-case dataset (n = 65; moderate–severe RHF events, n = 12). The apparent AUC was 0.854 and decreased to an optimism-corrected AUC of **0.773**. The optimism-corrected Brier score was 0.149, and the optimism-corrected calibration slope was **0.586**, indicating relevant optimism and potential overfitting. We added these findings to the manuscript and now explicitly state that the model is exploratory and not ready for clinical implementation.

**Changes made:**

- Added detailed bootstrap methods in **Methods – Statistical analysis**
- Added new **Table 6** after Table 5
- Added **S1 Fig** (Calibration plot) after Figure 2
- Revised **Results**, **Discussion**, **Limitations**, and **Conclusion**

**Location in revised files:** Methods, p. 9, lines 207–217; Results (Table 6), p. 16, lines 327–329; Discussion, pp. 18–19, lines 390–405; Limitations, p. 20, lines 424–437; Conclusion, p. 21, lines 446–457; Supporting information, S1 Fig.

**Reviewer #2, major comment 2: Validation study versus new model development**

We agree. The revised manuscript maintains the external validation of established RHF scores as the primary objective. The five-variable Firth model is explicitly described as a **secondary exploratory analysis**. We removed wording implying superiority or definitive model development and replaced it with cautious language emphasizing hypothesis generation and the need for external validation. Table 5 now labels the model as “Exploratory five-variable Firth model (apparent performance).”

**Changes made:**

- Revised **Abstract**, **Introduction/Aim**, **Results**, **Discussion**, and **Conclusion**
- Updated labeling in **Table 5**

**Location in revised files:** Abstract, pp. 2-3, lines 18–51; Introduction/Aim, p. 3 and p. 10; Table 5, p. 16, lines 316–320; Discussion, pp. 18–19, lines 390–405; Conclusion, p. 21, lines 446–457.

**Reviewer #2, major comment 3: Postoperative EUROMACS and possible temporal bias**

We agree that postoperative EUROMACS is conceptually different from preoperative models. We clarified that the postoperative EUROMACS score includes cardiopulmonary bypass time, which is only available after LVAD implantation; therefore, it was analyzed separately as an early perioperative/dynamic risk marker. We no longer present it as directly comparable to purely preoperative scores and explicitly acknowledge susceptibility to temporal bias in the limitations.

**Changes made:**

- Revised **Methods – Right heart failure predictive risk models**
- Updated **Results** and **Discussion**
- Added statement in **Limitations**

**Location in revised files:** Abstract, pp. 2-3, lines 18–51; Methods, pp. 6-7, lines 147–165; Results, p. 16, lines 297–306; Discussion, p. 17, lines 365–380; Limitations, p. 20, lines 424–437.

**Reviewer #2, major comment 4: Limited sample size and cautious interpretation**

We agree. We strengthened the limitations and use cautious wording throughout the manuscript. We specifically state that the exploratory Firth model was fitted in 65 complete cases with only 12 events, that bootstrap correction showed relevant optimism, and that the model requires external validation in larger independent multicenter cohorts. We also avoid recommending clinical implementation.

**Changes made:**

- Strengthened cautious interpretation in **Abstract**, **Results**, **Discussion**, **Limitations**, and **Conclusion**

**Location in revised files:** Abstract, pp. 2-3, lines 18–51; Methods, pp. 7–8; Results (Table 6), p. 16; Discussion, pp. 18–19; Limitations, p. 20, lines 424–437; Conclusion, p. 21, lines 446–457.

**Additional Journal Requirements**

All journal requirements regarding style, Data Availability Statement (now with institutional non-author contact), and file naming have been addressed.

We hope that the revised manuscript adequately addresses all comments. We thank the Academic Editor and reviewers again for their constructive feedback.

Sincerely, **Marjolijn C. Sales, MD** Corresponding Author
