# Supplementary material for: Predicting the right! Validation of right heart failure predictive risk models after primary durable ventricular assist device implantation
Source: PLoS One. 2026 Sep 9;21(9):e0357025. doi: 10.1371/journal.pone.0357025 (PMC13557412; doi:10.1371/journal.pone.0357025)
Supplement: Peer review history

# Peer review: Decision Letter 0

**DOI:** 10.1371/journal.pone.0357025.r001

**Authors:** Francesco Curcio

7 Jun 2026

Dear Dr. Sales,

Thank you for submitting your manuscript to PLOS ONE. After careful consideration, we feel that it has merit but does not fully meet PLOS ONE’s publication criteria as it currently stands. Therefore, we invite you to submit a revised version of the manuscript that addresses the points raised during the review process.

In line with the reviewers’ comments, the study addresses an important and clinically relevant question; however, several methodological issues require further clarification before the manuscript can be considered for publication. In particular, please address concerns regarding the development and validation of the proposed Firth model, clarify the rationale and timing of the postoperative EUROMACS score in relation to the study outcome, and moderate the interpretation of the newly derived model, presenting it as exploratory and hypothesis-generating.

Please submit your revised manuscript by Jul 22 2026 11:59PM. If you will need more time than this to complete your revisions, please reply to this message or contact the journal office at plosone@plos.org. When you're ready to submit your revision, log on to https://www.editorialmanager.com/pone/ and select the 'Submissions Needing Revision' folder to locate your manuscript file.

- A letter that responds to each point raised by the academic editor and reviewer(s). You should upload this letter as a separate file labeled 'Response to Reviewers'.
- A marked-up copy of your manuscript that highlights changes made to the original version. You should upload this as a separate file labeled 'Revised Manuscript with Track Changes'.
- An unmarked version of your revised paper without tracked changes. You should upload this as a separate file labeled 'Manuscript'.

If you would like to make changes to your financial disclosure, please include your updated statement in your cover letter. Guidelines for resubmitting your figure files are available below the reviewer comments at the end of this letter.

If applicable, we recommend that you deposit your laboratory protocols in protocols.io to enhance the reproducibility of your results. Protocols.io assigns your protocol its own identifier (DOI) so that it can be cited independently in the future. For instructions see: https://journals.plos.org/plosone/s/submission-guidelines#loc-laboratory-protocols. Additionally, PLOS ONE offers an option for publishing peer-reviewed Lab Protocol articles, which describe protocols hosted on protocols.io. Read more information on sharing protocols at https://plos.org/protocols?utm_medium=editorial-email&utm_source=authorletters&utm_campaign=protocols.

As the corresponding author, your ORCID iD is verified in the submission system and will appear in the published article. PLOS supports the use of ORCID, and we encourage all coauthors to register for an ORCID iD and use it as well. Please encourage your coauthors to verify their ORCID iD within the submission system before final acceptance, as unverified ORCID iDs will not appear in the published article. *Only*  the individual author can complete the verification step; PLOS staff *cannot*  verify ORCID iDs on behalf of authors.

We look forward to receiving your revised manuscript.

Kind regards,

Francesco Curcio, M.D., Ph.D.

Academic Editor

PLOS One

**Journal Requirements:**

When submitting your revision, we need you to address these additional requirements.

1. Please ensure that your manuscript meets PLOS ONE's style requirements, including those for file naming. The PLOS ONE style templates can be found at

https://journals.plos.org/plosone/s/file?id=wjVg/PLOSOne_formatting_sample_main_body.pdf and

https://journals.plos.org/plosone/s/file?id=ba62/PLOSOne_formatting_sample_title_authors_affiliations.pdf

2. In the online submission form you indicate that your data is not available for proprietary reasons and have provided a contact point for accessing this data. Please note that your current contact point is a co-author on this manuscript. According to our Data Policy, the contact point must not be an author on the manuscript and must be an institutional contact, ideally not an individual. Please revise your data statement to a non-author institutional point of contact, such as a data access or ethics committee, and send this to us via return email. Please also include contact information for the third party organization, and please include the full citation of where the data can be found.

3. If the reviewer comments include a recommendation to cite specific previously published works, please review and evaluate these publications to determine whether they are relevant and should be cited. There is no requirement to cite these works unless the editor has indicated otherwise.

**Additional Editor Comments:**

In line with the reviewers’ comments, the study addresses an important and clinically relevant question; however, several methodological issues require further clarification before the manuscript can be considered for publication. In particular, please address concerns regarding the development and validation of the proposed Firth model, clarify the rationale and timing of the postoperative EUROMACS score in relation to the study outcome, and moderate the interpretation of the newly derived model, presenting it as exploratory and hypothesis-generating.

[Note: HTML markup is below. Please do not edit.]

Reviewers' comments:

Reviewer's Responses to Questions

**Comments to the Author**

1. Is the manuscript technically sound, and do the data support the conclusions?

Reviewer #1: Yes

Reviewer #2: Partly

**********

2. Has the statistical analysis been performed appropriately and rigorously?

Reviewer #1: Yes

Reviewer #2: Yes

**********

3. Have the authors made all data underlying the findings in their manuscript fully available?

The PLOS Data policy

Reviewer #1: Yes

Reviewer #2: Yes

**********

4. Is the manuscript presented in an intelligible fashion and written in standard English?

Reviewer #1: Yes

Reviewer #2: Yes

**********

Reviewer #1: This manuscript presents an analysis of right heart failure (RHF) prediction models in a large real-world cohort of patients undergoing primary durable LVAD implantation. The study addresses an important clinical problem and provides valuable comparative insights into the performance of existing RHF risk scores while proposing a multimodal predictive approach.

Overall, the manuscript is well-structured, clinically relevant, and supported by a substantial dataset. The findings—particularly the limited performance of established risk models and the improved discrimination achieved by the proposed Firth-based model—are of interest to the field of advanced heart failure and mechanical circulatory support. However, several minor issues should be addressed to improve clarity, readability, and scientific rigor.

Major Comments

None. The study is methodologically sound, and the conclusions are generally supported by the data.

Minor Comments

1. Language and Formatting

The manuscript would benefit from careful language editing to correct typographical errors, spacing issues, and inconsistencies.

Some sentences are difficult to follow due to formatting artifacts and should be streamlined for clarity.

Ensure consistent terminology throughout (e.g., RHF, RVF, LVAD).

2. Clarity of Results Presentation

When reporting AUC values, consider consistently providing confidence intervals to enhance interpretability.

The transition between discussion of individual scores and the proposed model could be made smoother to improve readability.

3. Description of Modified Utah Score

The rationale for modifying the Utah score by including ECMO and Impella® devices should be clarified further.

Please specify whether this modification has been validated elsewhere or is exploratory.

4. Statistical Methods

Provide a brief justification for the use of Firth-penalized logistic regression (e.g., addressing small-sample bias or separation issues).

Clarify how variables were selected for inclusion in the 5-variable model.

5. Interpretation of Hemodynamic Parameters

The finding that commonly used hemodynamic parameters were not predictive is interesting but deserves slightly deeper discussion.

Consider elaborating on potential physiological explanations or measurement limitations.

6. Limitations Section

While limitations are appropriately acknowledged, consider explicitly mentioning potential selection bias due to single-center design.

Clarify whether missing data were present and how they were handled.

7. Future Directions

The discussion of machine learning approaches is relevant but somewhat general. If possible, briefly relate this to your dataset or suggest specific applications.

Conclusion

The manuscript provides meaningful insights into the limitations of current RHF prediction models and supports the use of a multimodal, integrative approach. With minor revisions addressing language, clarity, and methodological detail, the manuscript will be suitable for publication.

Reviewer #2: The study addresses a clinically relevant question: external validation of post-LVAD right heart failure (RHF) prediction models remains an important and unresolved issue. In addition, the manuscript is reasonably well written, the narrative is coherent, and the real-world dataset is interesting. However, there are several methodological and statistical concerns that would likely prevent direct acceptance in PLOS ONE.

The strengths of the manuscript include: a consecutively collected and reasonably well-characterized cohort; use of a contemporary INTERMACS definition of RHF; direct comparison of historical prediction scores; appropriate use of Firth regression given the small number of events; clinically meaningful outcomes.

However, there are several important limitations.

1. Major issue: overfitting and self-derived model. The authors propose a new “5-variable Firth model” with an AUC of 0.854, but:only 21 moderate-severe RHF events were available; the model was both derived and tested within the same cohort;

no bootstrap validation, internal validation, cross-validation, or calibration analysis was performed.

This is the main methodological concern. The reported AUC of 0.854 is likely optimistic and may not generalize well. The manuscript itself acknowledges the risk of overfitting.

I would strongly recommend that the authors include bootstrap optimism correction;

2. Conceptual inconsistency: validation study versus new score development The title and stated objective describe a validation study of existing scores. However, the manuscript then shifts toward development of a new predictive model.

This creates a conceptual and editorial issue: either this is an external validation study; or it is a model development study.

Currently, it appears to be an unfocused hybrid of both.

I would suggest:

reducing the emphasis on the newly derived model;

presenting it as an exploratory, hypothesis-generating analysis;

avoiding strong claims regarding superiority over existing scores.

3. Important methodological concern: use of the postoperative EUROMACS score

There is a significant methodological concern regarding comparison of a “postoperative EUROMACS score” against postoperative RHF outcomes.

This raises the possibility of temporal bias or information leakage:

a postoperative score is inherently advantaged;

it is not directly comparable to preoperative prediction models.

The authors should clarify:

the exact timing of score calculation;

whether variables were collected before RHF onset;

the rationale for including postoperative scores in the comparison.

Given a single-center cohort of only 103 patients, this conclusion appears too strong and should be substantially toned down.

Limited sample size

The study includes 103 total patients and only 21 events, which is relatively small for:

validating multiple prediction scores;

developing a new multivariable prediction model.

This requires cautious interpretation.

**********

what does this mean?). If published, this will include your full peer review and any attached files.

If you choose “no”, your identity will remain anonymous but your review may still be made public.

**Do you want your identity to be public for this peer review?**  For information about this choice, including consent withdrawal, please see our Privacy Policy

Reviewer #1: No

Reviewer #2: No

**********

[NOTE: If reviewer comments were submitted as an attachment file, they will be attached to this email and accessible via the submission site. Please log into your account, locate the manuscript record, and check for the action link "View Attachments". If this link does not appear, there are no attachment files.]

To ensure your figures meet our technical requirements, please review our figure guidelines: https://journals.plos.org/plosone/s/figures

You may also use PLOS’s free figure tool, NAAS, to help you prepare publication quality figures: https://journals.plos.org/plosone/s/figures#loc-tools-for-figure-preparation.

NAAS will assess whether your figures meet our technical requirements by comparing each figure against our figure specifications.

# Author comment: Author response to Decision Letter 1

**DOI:** 10.1371/journal.pone.0357025.r002

22 Jul 2026

Dear Academic Editor and Reviewers,

We thank the Academic Editor and both reviewers for their careful evaluation of our manuscript and for the constructive comments. We have revised the manuscript substantially to clarify the methodological framework, moderate the interpretation of the exploratory Firth model, add bootstrap internal validation and calibration analyses, clarify the timing and role of the postoperative EUROMACS score, and improve language, formatting, and consistency throughout the manuscript. We also completed S4 Table in the Supporting information. All changes are highlighted in the marked-up manuscript and incorporated in the clean revised manuscript. Page and line numbers below refer to the clean revised manuscript unless otherwise stated.

Academic Editor Comments

Editor comment 1: Firth model development, internal validation, and interpretation

We agree with the Academic Editor. We revised the manuscript to clearly distinguish the primary external validation of established RHF scores from the secondary exploratory analysis of the five-variable Firth model. We added bootstrap internal validation using 2,000 bootstrap resamples and now report optimism-corrected discrimination and calibration metrics. The apparent AUC of the exploratory model was 0.854 and decreased to an optimism-corrected AUC of 0.773; the optimism-corrected calibration slope was 0.586, indicating relevant optimism and potential overfitting. We therefore no longer describe the model as superior or clinically ready and consistently present it as exploratory and hypothesis-generating.

Changes made:

• Revised Abstract (Results and Conclusions)

• Added bootstrap description in Methods – Statistical analysis (new paragraph after the description of the Firth model)

• Added new Table 6 (Bootstrap internal validation) placed directly after Table 5 in the Results section

• Added reference to new S1 Fig (Calibration plot) after Figure 2

• Revised the paragraph discussing the Firth model in the Discussion

• Strengthened the Limitations section

Location in revised files: Abstract, p. 2, lines 18–51; Methods–Statistical analysis, pp. 7-10, lines 166–228; Results (Table 5–6 and interpretation), pp. 16-17, lines 316–349; Discussion, pp. 18–19, lines 370–405; Limitations, pp. 20-21, lines 424–444; Conclusion, p. 21, lines 447–457; Supporting information, S1 Fig., S2-S4 Table.

Editor comment 2: Timing and rationale of postoperative EUROMACS

We agree that the postoperative EUROMACS score is conceptually different from purely preoperative scores because it includes cardiopulmonary bypass time, which is only available after LVAD implantation. We clarified that postoperative EUROMACS was analyzed separately as an early perioperative/dynamic risk marker and should not be interpreted as a purely preoperative prediction model. We also added cautious language about temporal bias and its limited comparability with preoperative scores.

Changes made:

• Revised Methods – Right heart failure predictive risk models

• Updated Results – Model performance section

• Added explanation in Discussion and Limitations

Location in revised files: Abstract, pp. 2-3, lines 28–36 and 46–51; Methods–Right heart failure predictive risk models, pp. 6-7, lines 147–165; Results–Model performance, pp. 15-16, lines 308–315; Discussion, p. 18, lines 366–380; Limitations, p. 20, lines 424–437; Conclusion, p. 21, lines 446–457.

Editor comment 3: Data availability contact point

We revised the Data Availability Statement to provide a non-author institutional contact. The revised statement directs data requests to the Ethics Committee of the Medical Faculty of RWTH Aachen University and includes institutional contact information and the ethics approval number.

Changes made:

• Updated Data Availability Statement

Location in revised files: Data Availability Statement

Reviewer #1 Comments

Reviewer #1, minor comment 1: Language and formatting

We thank the reviewer. We revised the manuscript throughout for clarity, corrected typographical and spacing issues, and standardized terminology. We use RHF for right heart failure, RVF only when referring to the original names of published scores, and LVAD consistently. We also changed “5-variables” to “five-variable” and standardized “moderate–severe RHF”.

Changes made:

• Language and terminology edits throughout the manuscript

• Standardized figure and Supporting information captions

Location in revised files: Abstract, p. 2, lines 18–51; Methods–Right heart failure predictive risk models, pp. 6-7, lines 147–165; Figure 1 caption, p. 17, lines 345–350; Figure 2 caption, p. 17, lines 357–359; Supporting information captions.

Reviewer #1, minor comment 2: AUC confidence intervals and transition to exploratory model

We agree. Table 5 already reports AUC values with 95% confidence intervals. We revised the Results and Discussion to better separate validation of established scores from the secondary exploratory Firth model. We added Table 6 with bootstrap internal validation and calibration metrics, which provides a clearer transition from apparent to optimism-corrected performance. We also completed S4 Table in the Supporting information.

Changes made:

• Revised Results and Discussion for clearer separation of topics

• Added new Table 6 after Table 5

• Completed S4 Table in the Supporting information

Location in revised files: Results (Table 5–6), p. 16, lines 316–329; Discussion, pp. 18–19, lines 365–405; Supporting information, completed S4 Table.

Reviewer #1, minor comment 3: Modified Utah score

We agree. We clarified that the original Utah score includes intra-aortic balloon pump support, whereas ECMO and Impella® are now more commonly used as temporary MCS in our center. We explicitly state that the modified Utah score has not been externally validated and was analyzed only as an exploratory sensitivity analysis.

Changes made:

• Added clarification in Methods – Right heart failure predictive risk models

• Updated Discussion

Location in revised files: Methods, p. 7, lines 160–165; Discussion, pp. 18-19, lines 381–389; Supporting information, S4 Table.

Reviewer #1, minor comment 4: Statistical methods

We expanded the statistical methods section. We explain that standard logistic regression showed separation tendencies and convergence warnings due to the small event-to-variable ratio. Firth bias-reduced penalized logistic regression was therefore used to reduce small-sample bias and improve estimate stability. We also clarified how variables were selected for the final exploratory model and explicitly state that the model was not intended to represent a validated clinical score.

Changes made:

• Expanded justification for Firth regression in Methods – Statistical analysis

• Clarified variable selection process

Location in revised files: Methods–Statistical analysis, pp. 7-9, lines 166–206; Results–Multivariable modelling, p. 15, lines 291–301.

Reviewer #1, minor comment 5: Hemodynamic parameters

We agree and expanded the Discussion. We now explain that invasive hemodynamic measurements in advanced heart failure are affected by loading conditions, temporary MCS, ventilatory support, vasoactive medication, and volume status. We also note that static resting measurements may not capture right ventricular reserve or the dynamic response to LVAD-induced preload shifts, and that missingness and limited event number reduced statistical power.

Changes made:

• Expanded discussion of hemodynamic parameters in the Discussion section

Location in revised files: Discussion, pp. 19-20, lines 406–416.

Reviewer #1, minor comment 6: Limitations

We agree. We revised the Limitations section to explicitly mention single-center selection bias, limited generalizability, missing data, complete-case analysis (n = 65 with only 12 events), absence of imputation, and lack of external validation of the exploratory model.

Changes made:

• Strengthened Limitations section with more specific points

Location in revised files: Methods–Statistical analysis, p. 7, lines 169–173; Limitations, p. 20, lines 424–437.

Reviewer #1, minor comment 7: Future directions

We revised the future-directions paragraph to make it more specific. We now state that larger multicenter datasets are required for robust internal-external validation, calibration assessment, and evaluation of dynamic perioperative variables, advanced imaging, biomarkers, or explainable machine-learning approaches. We emphasize that such approaches require transparent reporting, calibration, and external validation before clinical implementation.

Changes made:

• Made future research paragraph more specific and actionable

Location in revised files: Future research paragraph, pp. 20-21, lines 438–444.

Reviewer #2 Comments

Reviewer #2, major comment 1: Overfitting and self-derived model

We fully agree with this concern. We performed bootstrap internal validation using 2,000 resamples. The exploratory Firth model was fitted in the complete-case dataset (n = 65; moderate–severe RHF events, n = 12). The apparent AUC was 0.854 and decreased to an optimism-corrected AUC of 0.773. The optimism-corrected Brier score was 0.149, and the optimism-corrected calibration slope was 0.586, indicating relevant optimism and potential overfitting. We added these findings to the manuscript and now explicitly state that the model is exploratory and not ready for clinical implementation.

Changes made:

• Added detailed bootstrap methods in Methods – Statistical analysis

• Added new Table 6 after Table 5

• Added S1 Fig (Calibration plot) after Figure 2

• Revised Results, Discussion, Limitations, and Conclusion

Location in revised files: Methods, p. 9, lines 207–217; Results (Table 6), p. 16, lines 327–329; Discussion, pp. 18–19, lines 390–405; Limitations, p. 20, lines 424–437; Conclusion, p. 21, lines 446–457; Supporting information, S1 Fig.

Reviewer #2, major comment 2: Validation study versus new model development

We agree. The revised manuscript maintains the external validation of established RHF scores as the primary objective. The five-variable Firth model is explicitly described as a secondary exploratory analysis. We removed wording implying superiority or definitive model development and replaced it with cautious language emphasizing hypothesis generation and the need for external validation. Table 5 now labels the model as “Exploratory five-variable Firth model (apparent performance).”

Changes made:

• Revised Abstract, Introduction/Aim, Results, Discussion, and Conclusion

• Updated labeling in Table 5

Location in revised files: Abstract, pp. 2-3, lines 18–51; Introduction/Aim, p. 3 and p. 10; Table 5, p. 16, lines 316–320; Discussion, pp. 18–19, lines 390–405; Conclusion, p. 21, lines 446–457.

Reviewer #2, major comment 3: Postoperative EUROMACS and possible temporal bias

We agree that postoperative EUROMACS is conceptually different from preoperative models. We clarified that the postoperative EUROMACS score includes cardiopulmonary bypass time, which is only available after LVAD implantation; therefore, it was analyzed separately as an early perioperative/dynamic risk marker. We no longer present it as directly comparable to purely preoperative scores and explicitly acknowledge susceptibility to temporal bias in the limitations.

Changes made:

• Revised Methods – Right heart failure predictive risk models

• Updated Results and Discussion

• Added statement in Limitations

Location in revised files: Abstract, pp. 2-3, lines 18–51; Methods, pp. 6-7, lines 147–165; Results, p. 16, lines 297–306; Discussion, p. 17, lines 365–380; Limitations, p. 20, lines 424–437.

Reviewer #2, major comment 4: Limited sample size and cautious interpretation

We agree. We strengthened the limitations and use cautious wording throughout the manuscript. We specifically state that the exploratory Firth model was fitted in 65 complete cases with only 12 events, that bootstrap correction showed relevant optimism, and that the model requires external validation in larger independent multicenter cohorts. We also avoid recommending clinical implementation.

Changes made:

• Strengthened cautious interpretation in Abstract, Results, Discussion, Limitations, and Conclusion

Location in revised files: Abstract, pp. 2-3, lines 18–51; Methods, pp. 7–8; Results (Table 6), p. 16; Discussion, pp. 18–19; Limitations, p. 20, lines 424–437; Conclusion, p. 21, lines 446–457.

Additional Journal Requirements

All journal requirements regarding style, Data Availability Statement (now with institutional non-author contact), and file naming have been addressed.

We hope that the revised manuscript adequately addresses all comments. We thank the Academic Editor and reviewers again for their constructive feedback.

Sincerely, Marjolijn C. Sales, MD Corresponding Author

Attachment Submitted filename: PONE-D-26-07516_Response_to_reviewers.docx

# Peer review: Decision Letter 1

**DOI:** 10.1371/journal.pone.0357025.r003

**Authors:** Francesco Curcio

11 Aug 2026

Predicting the right! Validation of right heart failure predictive risk models after primary durable ventricular assist device implantation

PONE-D-26-07516R1

Dear Dr. Sales,

We’re pleased to inform you that your manuscript has been judged scientifically suitable for publication and will be formally accepted for publication once it meets all outstanding technical requirements.

Within one week, you’ll receive an e-mail detailing the required amendments. When these have been addressed, you’ll receive a formal acceptance letter and your manuscript will be scheduled for publication.

An invoice will be generated when your article is formally accepted. Please note, if your institution has a publishing partnership with PLOS and your article meets the relevant criteria, all or part of your publication costs will be covered. Please make sure your user information is up-to-date by logging into Editorial Manager at Editorial Manager® and clicking the ‘Update My Information' link at the top of the page. For questions related to billing, please contact billing support.

If your institution or institutions have a press office, please notify them about your upcoming paper to help maximize its impact. If they’ll be preparing press materials, please inform our press team as soon as possible -- no later than 48 hours after receiving the formal acceptance. Your manuscript will remain under strict press embargo until 2 pm Eastern Time on the date of publication. For more information, please contact onepress@plos.org.

Kind regards,

Francesco Curcio, M.D., Ph.D.

Academic Editor

PLOS One

Additional Editor Comments (optional):

The authors have adequately addressed the reviewers’ concerns, and the manuscript has been substantially improved. In its current form, it merits publication in PLOS ONE.

Reviewers' comments:

Reviewer's Responses to Questions

**Comments to the Author**

Reviewer #1: All comments have been addressed

Reviewer #2: All comments have been addressed

**********

2. Is the manuscript technically sound, and do the data support the conclusions?

Reviewer #1: Yes

Reviewer #2: Yes

**********

3. Has the statistical analysis been performed appropriately and rigorously?

Reviewer #1: Yes

Reviewer #2: Yes

**********

4. Have the authors made all data underlying the findings in their manuscript fully available?

The PLOS Data policy

Reviewer #1: Yes

Reviewer #2: Yes

**********

5. Is the manuscript presented in an intelligible fashion and written in standard English?

Reviewer #1: Yes

Reviewer #2: Yes

**********

Reviewer #1: No further comments. All questions arised have been answered. The manuscript merit to be published in PlosOne.

Reviewer #2: After reviewing the revised manuscript and the detailed response to the reviewers, I believe that the authors have addressed the major methodological concerns raised during the previous review. In particular, they have:

clearly distinguished the primary validation study from the secondary exploratory Firth model;

added appropriate bootstrap internal validation and calibration analyses;

substantially moderated the interpretation of the exploratory model;

clarified the role of the postoperative EUROMACS score and its temporal limitations;

strengthened the Discussion and Limitations sections;

improved the statistical methods and reporting throughout the manuscript; and

considerably improved the language and overall presentation.

The manuscript is now more balanced, transparent, and appropriately cautious regarding the exploratory nature of the proposed model. The additional internal validation demonstrates awareness of the risk of overfitting and avoids overstating the clinical applicability of the findings.

I have only a few minor comments:

In the Discussion, the authors may further emphasize that the optimism-corrected calibration slope (0.586) indicates substantial overfitting, reinforcing that external validation is essential before any clinical application.

Some minor typographical and stylistic inconsistencies remain and should be corrected during copyediting, although the English has improved substantially compared with the previous version.

Overall, I believe the authors have satisfactorily addressed the reviewers' concerns, and the manuscript is now suitable for publication after minor revision.

**********

what does this mean?). If published, this will include your full peer review and any attached files.

If you choose “no”, your identity will remain anonymous but your review may still be made public.

**Do you want your identity to be public for this peer review?**  For information about this choice, including consent withdrawal, please see our Privacy Policy

Reviewer #1: No

Reviewer #2: No

**********

# Editor report: Acceptance letter

**DOI:** 10.1371/journal.pone.0357025.r004

**Authors:** Francesco Curcio

PONE-D-26-07516R1

PLOS One

Dear Dr. Sales,

I'm pleased to inform you that your manuscript has been deemed suitable for publication in PLOS One. Congratulations! Your manuscript is now being handed over to our production team.

At this stage, our production department will prepare your paper for publication. This includes ensuring the following:

* All references, tables, and figures are properly cited

* All relevant supporting information is included in the manuscript submission,

* There are no issues that prevent the paper from being properly typeset

You will receive further instructions from the production team, including instructions on how to review your proof when it is ready. Please keep in mind that we are working through a large volume of accepted articles, so please give us a few days to review your paper and let you know the next and final steps.

Lastly, if your institution or institutions have a press office, please let them know about your upcoming paper now to help maximize its impact. If they'll be preparing press materials, please inform our press team within the next 48 hours. Your manuscript will remain under strict press embargo until 2 pm Eastern Time on the date of publication. For more information, please contact onepress@plos.org.

You will receive an invoice from PLOS for your publication fee after your manuscript has reached the completed accept phase. If you receive an email requesting payment before acceptance or for any other service, this may be a phishing scheme. Learn how to identify phishing emails and protect your accounts at https://explore.plos.org/phishing.

If we can help with anything else, please email us at customercare@plos.org.

Thank you for submitting your work to PLOS One and supporting open access.

Kind regards,

PLOS One Editorial Office Staff

on behalf of

Dr. Francesco Curcio

Academic Editor

PLOS One
